# Is postoperative cognitive decline after cardiac surgery associated with plasma beta amyloid 1–42 levels?

**DOI:** 10.1186/s13019-022-01755-4

**Published:** 2022-01-16

**Authors:** Zrinka Požgain, Grgur Dulić, Goran Kondža, Siniša Bogović, Ivan Šerić, Dejan Hil, Bojan Trogrlić, Ana Bednjanić, Marina Perković-Kovačević, Ines Šahinović

**Affiliations:** 1 Department of Abdominal Surgery, University Hospital Centre Osijek, J. Huttlera 4, 31000 Osijek, Croatia; 2 Department of Cardiac Surgery, University Hospital Centre Osijek, J. Huttlera 4, 31000 Osijek, Croatia; 3 Department of Psychiatry, University Hospital Centre Osijek, J. Huttlera 4, 31000 Osijek, Croatia; 4 Department of Clinical Laboratory Diagnostics, University Hospital Centre Osijek, J. J. Strossmayer University of Osijek, J. Huttlera 4, Osijek, Croatia; 5 Medical Faculty Osijek, J. J. Strossmayer University of Osijek, Cara Hadrijana 10E, 31000 Osijek, Croatia

**Keywords:** Amyloid fibrils, Cognitive dysfunction, Coronary artery bypass surgery

## Abstract

**Background:**

Postoperative cognitive decline following cardiac surgery is one of the frequently reported complications affecting postoperative outcome, characterized by impairment of memory or concentration. The aetiology is considered multifactorial and the research conducted so far has presented contradictory results. The proposed mechanisms to explain the cognitive decline associated with cardiac surgery include the neurotoxic accumulation of β-amyloid (Aβ) proteins similar to Alzheimer's disease. The comparison of coronary artery bypass grafting procedures concerning postoperative cognitive decline and plasmatic Aβ1-42 concentrations has not yet been conducted.

**Methods:**

The research was designed as a controlled clinical study of patients with coronary artery disease undergoing surgical myocardial revascularization with or without the use of a cardiopulmonary bypass machine. All patients completed a battery of neuropsychological tests and plasmatic Aβ1-42 concentrations were collected.

**Results:**

The neuropsychological test results postoperatively were significantly worse in the cardiopulmonary bypass group and the patients had larger shifts in the Aβ1-42 preoperative and postoperative values than the group in which off-pump coronary artery bypass was performed.

**Conclusions:**

The conducted research confirmed the earlier suspected association of plasmatic Aβ1-42 concentration to postoperative cognitive decline and the results further showed that there were less changes and lower concentrations in the off-pump coronary artery bypass group, which correlated to less neurocognitive decline. There is a lot of clinical contribution acquired by this research, not only in everyday decision making and using amyloid proteins as biomarkers, but also in the development and application of non-pharmacological and pharmacological neuroprotective strategies.

## Background

Postoperative cognitive dysfunction (POCD) is defined as a disorder in thought processes which effects cognition in terms of memory, comprehension, and attention, affecting normal functioning. Perioperative cognitive decline is predictive of long-term cognitive dysfunction, reduced quality of life, and increased mortality [1, 2].

Recently, POCD has been investigated in all fields of surgery, mostly in cardiac surgery, where the incidence is twice as high as in other specialities. POCD is defined and measured by standardized mental function tests and the decline has been noted even 5 years after surgery, in spite of cerebral recovery. Numerous studies investigated the aetiology and perioperative risk factors contributing to the development of POCD and significant differences were observed related to cerebral microemboli. The precise pathophysiologic mechanisms of these factors influencing POCD development have not yet been described, but the proposed mechanisms for explaining the cognitive decline associated with cardiac surgery include the neurotoxic accumulation of β-amyloid (Aβ) proteins similar to Alzheimer's disease (AD) [2–4]. Neuropathologic characteristics of Alzheimer's disease include the presence of extracellular plaques of Aβ proteins. The damage of overexpressed Aβ to memory, learning, and hippocampal volume has been related to acetylcholine synthesis and release in the central nervous system (CNS) since nicotine acetylcholine receptors have the key role in the neurotransmission and regulation of memory, consciousness, and learning. The brain-blood barrier (BBB) damage has been proposed as a possible cause of POCD [5–16]. Few studies have demonstrated changes of plasmatic and liquor concentrations of Aβ1-42 in patients with POCD, where the postoperative plasmatic concentration was lower and liquor concentration was higher, although the results are contradictory and diverse between the studies [8, 12].

In this study we wanted to investigate the association of plasma Aβ1-42 levels with POCD after cardiac surgery and compare the on-pump coronary artery bypass grafting (CABG) procedure to off-pump CABG regarding the issue.

## Methods

The research was designed as a controlled clinical study. The study group consisted of patients with coronary artery disease (CAD) undergoing surgical myocardial revascularization during the five year period (from 2016. to 2020.).

To reach the effect size of 0,8 and find the difference in continuous variables between the on-pump and the off-pump groups with significance level of 0,05 and the power of 0,80 the sample size was calculated to 54 patients (G*Power ver. 3.1.2).

### Ethics

The institutional ethics committee approval was granted, and written informed consent was obtained from all patients (University Hospital Centre Osijek Ethical Committee, February 25th, 2016, R2-1060/2020.). A short interview with every individual patient was conducted regarding their lifestyle habits and all the anamnestic and perioperative data was collected for analysis. The study was conducted according to the Helsinki Declaration of 1964 and all subsequent revisions.

### Inclusion/exclusion criteria

Inclusion criteria were male patients, with no prior CVA, carotid artery stenosis over 60% narrowing by carotid duplex ultrasonography scans or significant renal insufficiency, suitable for neuropsychological testing, and scheduled to undergo elective first-time myocardial revascularization surgery. The patients were all native Croatians with Croatian as their first language and with different educational standard allocated to groups in a random fashion. Patients still suffering from the effects of analgesia, pulmonary dysfunction, acute heart failure, fluid retention, pain, any other postoperative complication in the time of the postoperative testing were excluded from the study since all these factors would have a bearing on the patients' ability to perform a battery of fairly sophisticated neuropsychological tests.

### Neuropsychological testing

All patients completed a battery of three neuropsychological tests administered by a trained interviewer. The tests were administered at baseline and at one week after the surgery. The test battery consisted of the Rey Auditory Verbal Learning Test (RAVLT), the Bender visual-motoric gestalt test second edition (Bender gestalt II) and the Mattis Dementia Rating Scale (DRS). These tests apply to the Statement of Consensus on Assessment of Neurobehavioral Outcomes After Cardiac Surgery published in Ann Thorac Surg 1995 (3). The tests are registered for Croatia and are appliable to demographic and epidemiological characteristics of the study patients. The RAVLT test is a very commonly used measuring instrument for examining memory in the clinical population. It is intended to examine short-term and long-term verbal memory, new learning abilities, learning strategies, and sensitivity to proactive and retroactive inhibition in children and adults. The Bender gestalt II test is useful for examining gifted people, people classified as mentally retarded, and people with disabilities (specific learning difficulties, attention deficit/hyperactivity disorder (ADHD), severe emotional disorder, autism, AD). It is also useful in neuropsychological research, in detecting organic damage and the degree of development of the nervous system, and for assessing visual maturity, visual-motor integration, response style, reaction to frustration, error correction skills, motivation, and recall skills. The test provides a quick assessment of memory and shows how well a person encodes, stores, and finds data. The DRS is considered a very useful instrument to assess patients with dementia. The tasks are grouped into five subscales, each one evaluating different cognitive areas, namely: Attention, Initiation/Perseveration, Construction, Conceptualization, and Memory.

### Sampling

Patient's peripheral vein blood was drawn before the operation and 7 days after, in a 3 ml vacutainer containing the EDTA anticoagulant (BD Vacutainer, Becton, Dickinson and Company, Francklin Lakes, New Jersey, SAD), centrifuged at 350 rpm for 10 min and stored at − 70 °C. Plasma Aβ1-42 was measured using the Aβ1-42 Highly Sensitive ELISA (IBL International GmbH, Hamburg, Germany). The test is a solid phase sandwich enzyme-linked immunosorbent assay (ELISA) that uses anti-human Aβ (38–42) mouse monoclonal antibody purified by affinity chromatography to detect plasma Aβ1-42. 100 μL of a patient’s plasma sample and the Aβ1-42 standard were added to the wells, and the plate was incubated overnight at 4 °C. The plate was washed, the enzyme conjugate (HRP conjugated anti-human Aβ IgG) was added, and the plate was incubated for 60 min at 4 °C on the Thermoshake Incubator shaker (C. Gerhardt Company GmbH, Königswinter, Germany). After the incubation, the plate was washed, the substrate solution was added, and the plate was incubated for 30 min at room temperature in the dark. A stop solution (1 M sulphuric acid) was added to all wells and the plate was read at 450 nm on the ETI-Max 3000 ELISA processor (DiaSorin SpA, Saluggia, Italy).

### Operative procedure

The anaesthetic protocol was standard for all the patients, regardless of the operative procedure. The induction was started with sufentanil (0.5 mcg/kg + 0.5–1 mcg/kg/h), etomidate (0.2–0.3 mg/kg) and rocuronium (1 mg/kg + 0.4 mg/kg/h), followed by intubation and mechanical ventilation (50% O_2_, air, inhalational anaesthetic—sevorane 1–2%). In the CABG group, during the ECC, the anaesthetic was added (opioid and propofol 2–3 mg/kg/h with a relaxant).

Both on-pump and off-pump surgery procedures were performed in standard fashion. In all patients in the on-pump group systemic anticoagulation was achieved with the intravenous administration of 300 I.U./kg of unfractioned heparin and the anticoagulation was checked by activated clotting time (ACT) which has to be over 480 s. All on-pump patients were cooled to 34 °C and were given warm blood cardioplegia (Calafiore). Initial dose of cardioplegia was delivered in orthograde fashion through a cannula placed into the aortic root, after the aortic cross-clamp was applied. Typical initial dose was 10–15 ml/kg. Cardioplegia was repeated every 10–15 min in retrograde fashion through a cannula placed in the coronary sinus in the dose of 500 ml. Mean arterial pressure during CPB was 60–80 mmHg.

In all the patients in the off-pump group the anticoagulation was achieved with the intravenous administration of 180 I.U./kg of unfractioned heparin and anticoagulation was checked by ACT which has to be over 300 s. In off-pump group the heart was stabilized by using Starfish (Medtronic Inc, Minneapolis, USA) tissue stabilizers.

All venous grafts in both groups were made using partial occlusion clamp on the ascending aorta [3].

### Statistical analyses

The sample size was calculated using the G*Power version 3.1.2, Franz Faul, Kiel University, Germany. The data was analysed using the Shapiro-Wilkes and Mann–Whitney's tests. The Receiver Operating Characteristic (ROC) analysis was used for determining the optimal borderline value for the area under the curve (AUC). The Spearman's correlation factor was used to evaluate the correlations. The analysis was undertaken using the MedCalc Statistical Software version 19.0.5 (MedCalc Software bvba, Ostend, Belgium; https://www.medcalc.org; 2019) and IBM SPSS Statistics for Windows, version 17 (IBM Corp. Armonk, N.Y., USA), and a probability value of less than 0.05 was taken to indicate significance.

## Results

Participants in the research were 54 male patients undergoing surgical myocardial revascularization, 63% were undergoing on-pump CABG and 37% off-pump CABG. The median age was 65, significantly older in the off-pump group (*P* = 0.03).

Body mass index (BMI) was 28,5 kg/m^2^ median, ranging from 25 kg/m^2^ to 31.3 kg/m^2^, there was no significant difference considering the type of surgery or POCD. 83% of the patients declared as not paying attention to their diet.

98% of the patients have arterial hypertension and 43% diabetes, all of which have diabetes type II. 10/23 patients are using insulin therapy, 13/23 peroral antidiabetic drugs and 4/23 combined therapy and there were no significant differences between the groups.

The duration of anaesthesia was significantly longer in the on-pump CABG group, compared to the off-pump group (*P* = 0.006).

44% of the patients had intraoperative blood transfusion, significantly more in the CABG group—59% of the patients (Fishers' test, *P* = 0.01). There were no significant differences between the groups considering POCD.

81% of the patients were extubated the same day, meaning that the majority of the patients did not have prolonged mechanical ventilation time or ICU time and there were no significant differences between the groups.

All of the patients recovered from the surgery and were released from the hospital.

In the on-pump group, the absolute difference between the Aβ1-42 preoperative and postoperative values was significantly larger, with the median of 1.33 (IQR 0.39 to 3.51) (*P* = 0.02) (Table 1).

**Table 1:** Preoperative and postoperative Aβ1-42 values and absolute difference

	Median (25–75%)		Difference^†^	95% CI		P*
	CABG	OPCAB		From	To	
Aβ1-42						
Preoperative	1.89 (0.56–5.21)	1.18 (0.36–3.42)	− 0.63	− 2.16	0.12	0.09
Postoperative	2.82 (0.62–5.59)	1.24 (0.51–2.92)	− 0.93	− 2.78	0.08	0.09
Absolute difference preoperative and postoperative Aβ1-42	1.33 (0.39–3.51)	0.24 (0.135–0.88)	− 0.7	− 2.03	− 0.07	**0.02**

Bold typing was used for statistically significant results, meaning that P* was < 0.005

CABG, coronary artery bypass grafting; OPCAB, off-pump coronary artery bypass; CI, reliability spectrum

*Mann Whitney U test

^†^Hodges-Lehmann

While there were no significant differences between the on-pump CABG and off-pump groups in tracing and memory recollection before the operative procedure, the tracing results postoperatively were significantly worse in the on-pump CABG group, with the median of 32.5 (IQR 11 to 53) (*P* = 0.03).

The association of POCD with amyloid plasmatic concentrations, with absolute difference of amyloid concentrations preoperatively and postoperatively and with the duration of anaesthesia, was evaluated using the Spearman's correlation factor. In all the patients, plasmatic Aβ1-42 concentration after the operation was significantly and negatively connected to the conceptualisation domain (Rho = − 0.375) and to the cognitive functioning in general (Rho = − 0.324) according to the DRS scale, which means that the patients with higher Aβ1-42 postoperative values had worse results in the domain of conceptualisation and general cognitive functioning. In the on-pump CABG group, worse results in conceptualisation (Rho = − 0.472) and general cognitive functioning (Rho = − 0.362) were present in patients with higher postoperative Aβ1-42 values. In the construction domain, the results were worse if the absolute difference between plasmatic Aβ1-42 values was larger (Rho = − 0.339). In the off-pump group, worse results in the memory recollection domain (Rho = − 0.471) were present in patients with higher postoperative Aβ1-42 values. The longer duration of the anaesthesia was connected to worse results in the attention domain (Rho = − 0.509) in the off-pump group (Table 2).

**Table 2:** Domains with significant correlations of cognitive status to plasmatic Aβ1-42 concentrations, the absolute difference of Aβ1-42 before and after the operation and to the duration of anaesthesia (Spearman's correlation factor) in all patients and in the on-pump CABG and off-pump groups

Postoperatively	Spearman's correlation factor (P value)			
	Aβ1-42 Preop	Aβ1-42 Postop	Absolute difference	Anaesthesia duration
All patients				
Construction (DRS)	− 0.068 (0.63)	− 0.203 (0.14)	**−** **0.264 (0.04)**	0.049 (0.72)
Conceptualisation (DRS)	− 0.168 (0.22)	**−** **0.375 (0.01)**	− 0.18 (0.19)	0.125 (0.37)
Total (DRS)	− 0.114 (0.41)	**−** **0.324 (0.02)**	− 0.16 (0.25)	0.118 (0.40)
CABG				
Construction (DRS)	− 0.065 (0.71)	− 0.290 (0.10)	**−** **0.339 (0.04)**	0.121 (0.50)
Conceptualisation (DRS)	− 0.159 (0.37)	**−** **0.472 (< 0.001)**	− 0.254 (0.15)	0.105 (0.55)
Total (DRS)	− 0.085 (0.63)	**−** **0.362 (0.04)**	− 0.204 (0.25)	0.098 (0.58)
OPCAB				
Memory (BG)	− 0.331 (0.15)	**−** **0.471 (0.04)**	− 0.07 (0.77)	0.223 (0.34)
Attention (DRS)	0.015 (0.95)	− 0.180 (0.45)	− 0.245 (0.30)	**−** **0.509 (0.02)**

Bold typing was used for statistically significant results, meaning that P* was < 0.005

Using the ROC analysis, significant factors predicting worse postoperative neurocognitive status were the absolute difference in plasmatic Aβ1-42 values before and after the operation in the tracing domain (AUC = 0.680, *P* = 0.03) and the plasmatic Aβ1-42 values after the surgery in the conceptualisation domain of the DRS scale (AUC = 0.796, *P* < 0.001).

BMI, age, prothrombin time, urea, creatinine and ejection fraction were additionally tested for correlation to Aβ1-42 concentrations and absolute changes in concentrations. There were no significant differences except the correlation of BMI to smaller absolute change in Aβ1-42 plasmatic concentrations after the surgery (Table 3).

**Table 3:** The correlations of plasmatic Aβ1-42 concentrations and changes with age, BMI, ejection fraction, prothrombin time, urea and creatinine values

	Spearman's correlation Rho (*P* value)		
	Aβ1-42 before	Aβ1-42 after	Absolute difference
All the patients			
Age	0.038 (0.78)	0.087 (0.53)	− 0.113 (0.42)
BMI	0.145 (0.30)	0.186 (0.18)	0.237 (0.09)
Urea	0.135 (0.33)	0.051 (0.72)	− 0.001 (0.99)
Creatinin	0.249 (0.07)	0.158 (0.25)	0.111 (0.42)
Prothrombin time	0.081 (0.56)	0.008 (0.95)	0.199 (0.15)
Ejection fraction	− 0.132 (0.34)	0.126 (0.37)	0.092 (0.51)
CABG			
Age	− 0.056 (0.75)	0.030 (0.87)	− 0.247 (0.16)
BMI	− 0.043 (0.81)	0.064 (0.72)	0.075 (0.67)
Urea	0.064 (0.72)	− 0.102 (0.57)	− 0.114 (0.52)
Creatinin	0.086 (0.63)	− 0.017 (0.93)	− 0.097 (0.59)
Prothrombin time	− 0.020 (0.91)	− 0.110 (0.53)	0.149 (0.40)
Ejection fraction	− 0.166 (0.35)	0.145 (0.41)	− 0.014 (0.94)
OPCAB			
Age	0.249 (0.29)	0.401 (0.08)	0.270 (0.25)
BMI	**0.459 (0.04)**	0.342 (0.14)	**0.449 (0.04)**
Urea	0.196 (0.41)	0.254 (0.28)	0.136 (0.57)
Creatinin	0.409 (0.07)	0.348 (0.13)	0.410 (0.07)
Prothrombin time	0.176 (0.46)	0.116 (0.63)	0.222 (0.35)
Ejection fraction	− 0.246 (0.30)	− 0.168 (0.48)	0.062 (0.80)

Bold typing was used for statistically significant results, meaning that P* was < 0.005

CABG, coronary artery bypass grafting; OPCAB, off-pump coronary artery bypass; CI, reliability spectrum

*Mann Whitney U test

^†^Hodges-Lehmann

In the CABG group the median systolic function of left ventricle is 59% (49–61% interquartile range) which is significantly higher than the median ejection fraction of patients in the off-pump group (Mann Whitney U test, *P* = 0.04). 70.4% of the patients have ejection fraction higher than 50% and it was not significantly correlated to POCD (Table 3).

## Discussion

POCD following cardiac surgery is one of the frequently reported complications affecting postoperative outcome, characterized by the impairment of memory or concentration, detected by neuropsychological testing and clinically presenting with deficits in cognition and memory. According to the study that assessed neurological complications after CABG the incidence was 3%. The results of the studies are contradictory and POCD after cardiac surgery operations is still the topic of research. Patients who have undergone the off-pump CABG procedure had lower incidence of early POCD in 46% of the cases, while the results where similar to on-pump CABG patients after one year [3, 4]. Patients who have undergone valvular replacement had more POCD compared to patients who have undergone CABG. The patients with valvular disease were excluded from the research, considering the differences in the pathology and operative procedure. Although the risk of air and tissue debris embolism is obviously greater in valvular surgery and the higher incidence of POCD after valvular surgery than after CABG has been reported, currently available evidence with regard to cerebral desaturation and POCD are mostly confined to CABG [19].

The aetiology of POCD is considered multifactorial with the most commonly cited aetiologies being embolism and hypoperfusion related to CPB, affecting the cerebral oxygen supply–demand balance [19, 20] and pathophysiologic influences of the partial aortic clamp, inflammatory mediators, anaesthetics, atrial fibrillation, intra-aortic balloon pump (IABP) use, prolonged and repeated use of the extracorporeal circulation machine for cardiopulmonary bypass (CPB), inotrope use, ascendant aortic and carotid artery disease, previous cerebrovascular accident (CVA), age above 70, systolic hypertension, pulmonary disease, diabetes, and alcohol abuse [2–4].

In our research, we only included the male patients to get clearer results considering the gender-related neurotoxic effects. The male brain is more vulnerable to many toxic exposures than the female brain, with evidence suggesting this difference includes: greater neuroinflammatory response in males; reduced vulnerability to oxidative stress in females; and neuroprotective effects of oestrogen and progesterone, especially in the reduction of inflammation and oxidative stress [21].

POCD in cardiac surgery has been investigated in the last decades, giving contradictory results. Some studies showed that perioperative neurocognitive decline predicts long-term cognitive dysfunction [20, 22]. The use of the CPB machine is frequently suspected as contributing to the risk of POCD, because the CPB machine can cause embolization, as well as activate systemic inflammatory pathways. A study done on the Chinese population shows that although CPB increased the number of cerebral micro-emboli, it did not increase the incidence of POCD compared with the off-pump group, suggesting another aetiology for cognitive decline, perhaps by the activation of inflammatory pathways. Pro-inflammatory cytokines that are released in response to surgery include the tumour necrosis factor α (TNF-α) and interleukin (IL) IL-1β and IL-6, which can then induce the production and release of other pro-inflammatory cytokines, resulting in neuroinflammation leading to Aβ plaque formation, which is known to be associated with AD pathology [16]. Although not well understood, several potential activities have been discovered for Aβ, including the activation of kinase enzymes, protection against oxidative stress, regulation of cholesterol transport, functioning as a transcription factor, and anti-microbial activity (potentially associated with Aβ's pro-inflammatory activity) [5–16]. The damage of overexpressed Aβ to memory, learning, and hippocampal volume has been related to acetylcholine synthesis and release in the central nervous system (CNS). Nicotine acetylcholine receptors have the key role in the neurotransmission and regulation of memory, consciousness, and learning. The brain-blood barrier (BBB) damage caused by inflammatory mediators as a response to iatrogenic factors such as intraoperative cerebral hypoxia, hypocapnia, hypoperfusion with loss of autoregulation, cerebral emboli, or high glucose concentrations has been proposed as a possible cause of POCD [5–16]. The most recent publication showed that older adults with early cognitive dysfunction develop brain capillary damage associated with mural cell pericyte injury and BBB breakdown in the hippocampus irrespective of Aβ changes, suggesting that BBB breakdown is an independent, early biomarker of cognitive impairment unrelated to Aβ [17]. On the other hand, one of the physiological functions of Aβ is to control cholesterol transport and homeostasis, so the overproduction of Aβ blocks cholesterol trafficking, leading to neurodegeneration [18].

Most of the studies have found no significant difference comparing on-pump CABG to off-pump CABG regarding POCD. This could be further confirmation of the suggestions that many other factors such as inflammatory processes including sternotomy, heparin administration, and hemodynamic variations may be responsible for cognitive dysfunction [20].

We compared the on-pump and the off-pump procedure regarding both the POCD and the Aβ1-42 plasma concentrations and found that in the on-pump CABG group, the absolute difference in the Aβ1-42 preoperative and postoperative values was significantly larger and associated to cognitive decline. The ROC analysis showed that the absolute difference in plasmatic Aβ1-42 values before and after the operation and the plasmatic Aβ1-42 postoperative values are significant factors predicting worse postoperative neurocognitive status. We suggest that the use of CPB and the associated inflammatory processes contribute great deal to the POCD aetiology. Another argument for the benefit of off-pump surgery regarding POCD is the fact that the patients in the off-pump group were significantly older and yet had better results and less cognitive decline.

Since the off-pump group, although older, had better cognitive outcomes, we suggest that there is a relation to surgical factors. We can assume that these factors include the ECC affecting the patient’s inflammatory response leading to different cognitive outcomes and amyloid concentrations. Other possible factors could include the number of proximal anastomoses because of the partial clamping of the aorta although the partial clamping was used in all of the patients, cross clamping in CABG patients, mild hypothermia, etc.

Our results also showed the association of the anaesthesia duration to the POCD but only in the off-pump group, which is quite interesting and different from the results in the available literature. In the off-pump group, the duration of the anaesthesia was correlated to worse results in the DRS attention tests.

There are many studies in the available literature comparing the different types of anaesthesia to different cognitive outcomes with various and contradictive results. In our study, the anaesthetic protocol was standard for all the patients, there was combined intravenous and inhalational anaesthetics so there was no bias resulting from different types of anaesthesia.

Since various factors associated to anaesthesia such as mechanical ventilation time and ICU time could affect cerebral oxygen metabolism according to multiple studies with contradictory results, we also compared the groups considering mechanical ventilation and ICU time and there were no significant differences. Intraoperative blood transfusion was significantly more common in the CABG group but we still did not find significant difference between the group considering the POCD.

The possible confounding bias caused by BMI, diabetes, heart failure and left ventricular ejection fraction was excluded dividing the patients in subgroups and comparing them considering the POCD where there were no significant differences. The correlations of age, BMI, ejection fraction, prothrombin time, urea and creatinine to Aβ1-42 concentrations and absolute changes of the concentration were tested with Spearman’s correlation factor and while there were no significant results, the only significant result was the correlation of BMI to smaller absolute change in Aβ1-42 plasmatic concentrations after the surgery (Table 3). This result is interesting because it is not expected—it can be interpreted in a way that the patients with larger BMI (obese patients) had ‘better’ results in the plasmatic amyloid shift. While there was no correlation of BMI to POCD, this smaller shift in the plasmatic amyloid concentrations could mean that there are complex processes in the amyloid pathophysiology connected to metabolic reactions.

In the CABG group the median systolic function of left ventricle is 59% (49%-61% interquartile range) which is significantly higher than the median ejection fraction of patients in the off-pump group (Mann Whitney U test, *P* = 0.04). 70.4% of the patients have ejection fraction higher than 50% and it was not significantly correlated to POCD (Table 3). In our study there were no patients with an acute heart failure, it was part of the exclusion criteria.

The association of POCD with postoperative amyloid values implicates Aβ proteins in the pathogenesis of early POCD, at least at this particular time interval. The relationship of Aβ1-42 to POCD in the early postoperative period may have important clinical implications, such as using amyloid levels as a mechanism for screening the patients with higher risk after they have undergone the surgery procedure. This screening could additionally guide the surgeon in the postoperative recovery using amyloids as a biomarker for cognitive impairment after surgery. If the POCD does result from mechanisms related to AD, then these patients may provide a suitable group for testing prophylactic therapies against AD, because effects would be seen in a short period. Our results could also be used in the development and application of non-pharmacological and pharmacological neuroprotective strategies and might lead to the study of amyloid-specific interventions to prevent these long term post-surgical effects. Since this is the first study comparing the on-pump CABG and off-pump procedures regarding both the POCD and potential biomarkers, we believe our results show the advantage of the off-pump surgery in patients with higher neurological risks and contributes to further research. Efforts should also be made in developing techniques which would lessen the duration of both on-pump and off-pump procedures, since the duration of the anaesthesia seems to be correlated to worse neurocognitive decline in the off-pump group. Further studies are needed to continue the investigation of the factors influencing the cognitive decline and amyloid shifting, such as ECC variables, partial clamping and others that could be modified and in that way improve the surgical technique.

### Study limitations

We did not evaluate the binary outcomes of the data regarding the cognitive decline because the tests refer to different variables and cannot be compared in general, thus we believe we achieved more precise conclusions evaluating each test separately. This separate interpretation of the data limits the study in generalising the data. Another limitation is still not knowing the long term outcomes of the patients. Therefore, we are planning another study which would complete these early POCD data with the long term outcomes. The surgical procedures were performed by different surgeons so that might also be one of the limits, although there were no significant differences in operating time.

## Conclusion

This research confirmed the earlier suspected association of plasmatic Aβ1-42 concentration to POCD and the results further showed that there were less changes and lower amyloid protein concentration changes in the off-pump group, which correlated to less neurocognitive decline than in the on-pump CABG group. The off-pump procedure seems to be more protective for patients at greater neurological risk and efforts should be made in developing techniques for making both procedures shorter to shorten the duration of the anaesthesia and minimize the role it has in the POCD aetiology. Further research is needed regarding the use of amyloid proteins as biomarkers and developing treatments.

## Data Availability

The datasets generated and analysed during the current study are not publicly available due to the privacy of the patients and confidentiality of the Hospital information system but are available from the corresponding author on reasonable request.

## Abbreviations

Aβ: β-Amyloid
AD: Alzheimer's disease
ADHD: Attention deficit/hyperactivity disorder
AVLT: Auditory Verbal Learning Test
BBB: Brain-blood barrier
Bender gestalt II: Bender visual-motoric gestalt test second edition
CABG: Coronary artery bypass grafting
CAD: Coronary artery disease
CNS: Central nervous system
CPB: Cardiopulmonary bypass
CVA: Cerebrovascular accident
DRS: Mattis Dementia Rating Scale
ELISA: Enzyme-linked immunosorbent assay
IABP: Intra-aortic balloon pump
IL: Interleukin
OPCAB: Off-pump coronary artery bypass
POCD: Postoperative cognitive dysfunction
TNF- α: Tumour necrosis factor α
